# Evolution of the Gene Lineage Encoding the Carbon Dioxide Receptor in Insects

**DOI:** 10.1673/031.009.1901

**Published:** 2009-05-13

**Authors:** Hugh M. Robertson, Lauren B. Kent

**Affiliations:** Department of Entomology, University of Illinois at Urbana-Champaign, 505 S. Goodwin Ave., Urbana, IL 61801, USA

**Keywords:** olfaction, gustatory receptor, smell, intron evolution

## Abstract

A heterodimer of the insect chemoreceptors Gr21a and Gr63a has been shown to be the carbon dioxide receptor in *Drosophila melanogaster* (Meigen) (Diptera: Drosophilidae). Comparison of the genes encoding these two proteins across the 12 available drosophilid fly genomes allows refined definition of their N-termini. These genes are highly conserved, along with a paralog of Gr21a, in the *Anopheles gambiae, Aedes aegypti*, and *Culex pipiens* mosquitoes, as well as in the silk moth *Bombyx mori* and the red flour beetle *Tribolium castaneum*. In the latter four species we name these three proteins Gr1, Gr2, and Gr3. Intron evolution within this distinctive three gene lineage is considerable, with at least 13 inferred gains and 39 losses. Surprisingly, this entire ancient gene lineage is absent from all other available more basal insect and related arthropod genomes, specifically the honey bee, parasitoid wasp, human louse, pea aphid, waterflea, and blacklegged tick genomes. At least two of these species can detect carbon dioxide, suggesting that they evolved other means to do so.

## Introduction

Many insects are capable of sensitive detection of elevated levels of carbon dioxide (CO_2_) (e.g. Stange 1996; Stange and Stowe 1999). Perhaps most famously, many hematophagous insects and other arthropods such as mosquitoes (e.g. Gillies 1980; Bowen 1991), tsetse flies (e.g. Vale and Hall 1985; Gibson and Torr 1999), and ticks (e.g. Holscher et al. 1980) use elevated CO_2_ concentrations to locate their vertebrate hosts. Several moths employ CO_2_ gradients in evaluating floral quality (e.g. Stange et al. 1995; Thom et al. 2004), while social insects like honeybees and ants regulate potentially lethal CO_2_ concentrations in their social colonies (e.g. Seeley 1974; Kleineidam and Tautz 1996). The importance of carbon dioxide perception in other flies like *Drosophila melanogaster* (Meigen) (Diptera: Drosophilidae) has also been recognized (de Bruyne et al. 2001), where an increase in CO_2_ released by stressed flies elicits avoidance behavior (Suh et al. 2004), and with this came the potential to discover the molecular nature of the elusive carbon dioxide receptor. DmGr21a is expressed in the ab1C olfactory receptor neurons (ORNs) in the ab1 sensilla on the antennae of *D. melanogaster* that are sensitive to CO_2_ (de Bruyne et al. 2001; Suh et al. 2004), thereby implicating this member of the nominal gustatory receptor family within the insect chemoreceptor superfamily (Clyne et al. 2000; Scott et al. 2001; Dunipace et al. 2001; Robertson et al. 2003). Recently Jones et al. (2007) and Kwon et al. (2007) reported that the related receptor DmGr63a is also expressed in these neurons, and showed that both are required for detection of carbon dioxide when mis-expressed in an ORN without receptors. This breakthrough in identification of the heterodimeric nature of the carbon dioxide receptor in *Drosophila* flies has general implications, because this pair of Grs is highly conserved in the African malaria vector *Anopheles gambiae* mosquito, where they were named AgGr22 and AgGr24 (Hill et al. 2002). Indeed, Jones et al. (2007) show that AgGr22 and AgGr24 are also co-expressed in a set of ORNs, but this time in sensilla on the maxillary palps of *Anopheles* mosquitoes, the organ known to mediate their perception of carbon dioxide (e.g. Bowen 1991). Finally, Lu et al. (2007) show that this pair of receptors is sufficient for CO_2_ perception in this mosquito.

Here we describe the evolution of these two Gr genes and proteins, and a third related gene/protein that was lost from the drosophilid fly lineage, in the available endopterygote (holometabolous) insect genomes. This third gene is also expressed in the same set of ORNs in sensilla on the palps of *Anopheles* mosquitoes and greatly improves the sensitivity to CO_2_ when co-expressed with the other two proteins in *D. melanogaster* neurons (Lu et al. 2007). Remarkably, this entire three-gene lineage is not present in the available more basal hymenopteran genome sequences (honey bee and parasitoid wasp), nor is it present in any other available more basal insect or related arthropod genome sequences, despite the ability of at least two of these arthropods to detect carbon dioxide.

## Materials and Methods

The Gr21a and Gr63a orthologs were retrieved from the 12 *Drosophila* genome sequences available at FLYBASE and GENBANK, as were orthologs of the AgGr22-24 genes from the *Aedes aegypti, Bombyx mori*, and *Tribolium castaneum* genome assemblies, using TBLASTN searches. All other available unpublished draft arthropod genome sequences were also searched using TBLASTN, as were the entire sets of raw reads at the Trace Archive using the NCBI BLAST CLIENT software. Gene models were built manually in the text editor of PAUP*v4.0b10 (Swofford 2002). The gene models for BmGr1 and BmGr2 could not be confidently completed for their N-terminus because the expected upstream N-terminal coding exon(s) could not be identified due to the expected high divergence of the encoded amino acids. The N-terminal region of BmGr3 was constructed from a combination of the available contigs from both the Japanese and Chinese genome assemblies, both of which have frameshifting errors. The amino acid sequences are available in the Supplement.

Proteins were aligned in CLUSTALX (Jeanmougin et al. 1998) using default parameters, and phylogenetic analysis was performed using corrected distance methods. Corrected distances were calculated in TREE-PUZZLE v5.0 (Schmidt et al. 2002) using the BLOSUM62 amino acid matrix in their maximum likelihood model, and distance trees were estimated in PAUP*v4.0b10 using tree-bisection-and-reconnection branch swapping. Support for branches was obtained from 1000 bootstrap replications of uncorrected distance analysis. Inferred intron gains and losses were mapped onto the phylogenetic tree using simple parsimony and equal weighting of gains and losses. Kyte-Doolittle hydropathy plots were produced in DNA Strider v1.1 (Marck 1988).

## Results and Discussion

### Gr21a/63a in other drosophilid flies

Highly conserved orthologs of the DmGr21a and DmGr63a genes are present in the *Drosophila pseudoobscura* genome sequence (Richards et al. 2005; Robertson 2009) and the other 10 newly available *Drosophila* genome sequences (Drosophila 12 Genomes Consortium 2007), as expected from the presence of conserved orthologs in *An. gambiae* (Hill et al. 2002). Alignments of the genes and their encoded protein products in these 12 *Drosophila* species allowed refinement of their uncertain N-termini. Both DmGr21a and DmGr63a have extended potential ORFs in their first coding exons beyond a potential start codon that is conserved in all the other species, adding 47 and 23 additional amino acids respectively. These N-terminal extensions were originally annotated as part of these proteins in the absence of comparative information, and at least for DmGr21a are supported by the existence of a single full-length cDNA clone from the Berkeley Drosophila Genome Project (BDGP) (GenBank BT025007.1), which has a 674 bp 5′ UTR in front of this most upstream available start codon.

For Gr21a this possible 47 amino acid extension is present in the sibling species *D. simulans* and *D. sechellia*, as well as the more distantly related *D. yakuba*. There is, however, a single base pair insertional frameshift in this extension in *D. erecta* (confirmed by all eight reads that cover this region), which eliminates the possibility of this N-terminal extension being functional in this species. No such extension is possible for *D. ananassae* in which this upstream sequence is considerably diverged, nor is any extension possible for the more divergent *D. willistoni, D. mojavensis, D. virilis*, or *D. grimshawi* genes. Remarkably, an N-terminal extension of 55 amino acids is also possible for *D. pseudoobscura*, however the amino acid sequence is completely different from the *D. melanogaster* extension. Furthermore, although the DNA sequence in this region is similar in the sibling species *D. persimilis*, a 2 bp insertional frameshift eliminates the possibility of it being functional in this species, and suggests that it is unlikely to be functional in *D. pseudoobscura* either.

For Gr63a the possible 23 amino acid N-terminal extension is similarly shared with the sibling species *D. simulans* and *D. sechellia*, as well as both *D. yakuba* and *D. erecta*, however in *D. simulans* there is an alternate allele represented by two of the ten reads that cover the region which has a four-base frameshifting deletion in this extension. Again, no extension is possible for the more divergent *D. ananassae, D. willistoni, D. mojavensis, D. virilis*, or *D. grimshawi* genes, however once again *D. pseudoobscura* and *D. persimilis* share a possible extension of 11 amino acids of completely different sequence from DmGr63a.

Given the high conservation of the rest of these proteins, we believe these N-terminal extensions are not present in these proteins in these flies, thus the true N-terminus of DmGr21a is likely to be MSFWAV and that of DmGr63a is MANYYR (full amino acid sequences for all species are in the Appendix). If correct, this would require that the translation machinery ignore the first two available AUG start codons in the extended ORF of DmGr21a, but this seems feasible as it must already ignore another 14 potential AUG start codons in the 674 bp 5′ UTR. Thus two of the versions of this protein available in GenBank likely have incorrectly elongated N-termini. Accession ABE01237.1 encoding “IP03362p” is from the BDGP cDNA with 47 additional amino acids on the N-terminus, while ABK97615.1 encoding “gustatory receptor 21a” is from Jones et al. (2007) and appears not to represent an experimentally determined cDNA but rather is a conceptual coding sequence from the start to stop codons and encodes eight additional amino acids on the N-terminus. A third version in GenBank, represented by accession AAF51461.2, is our conceptual CDS from FlyBase, “CG13948-PA”. Unfortunately the only way to confirm our inference would be to obtain N-terminal peptide sequence from these proteins as they are expressed in fly antennal neurons. It is also possible that these N-terminal extensions are sometimes present and/or do not affect function of the proteins, after all, the slightly extended version was employed by Jones et al. (2007) in their experiments identifying these proteins as a heterodimeric receptor for carbon dioxide.

Another somewhat unusual feature of these *Drosophila* genes is the different introns in the Gr21a lineage (the Gr63a genes in these drosophilids all have the same two introns). The divergent lineage of *D. mojavensis/virilis/grimshawi*, as well as *D. willistoni* and the *D. pseudoobscura/persimilis* pair have no introns, while *D. ananassae* has one intron, and the *melanogaster* subgroup species (*D. yakuba/erecta/simulans/sechellia/melanogaster*) have two more introns. This interesting intron evolution is explored further below.

### Three genes in mosquitoes, silk moth, and flour beetle

**Figure 1.**
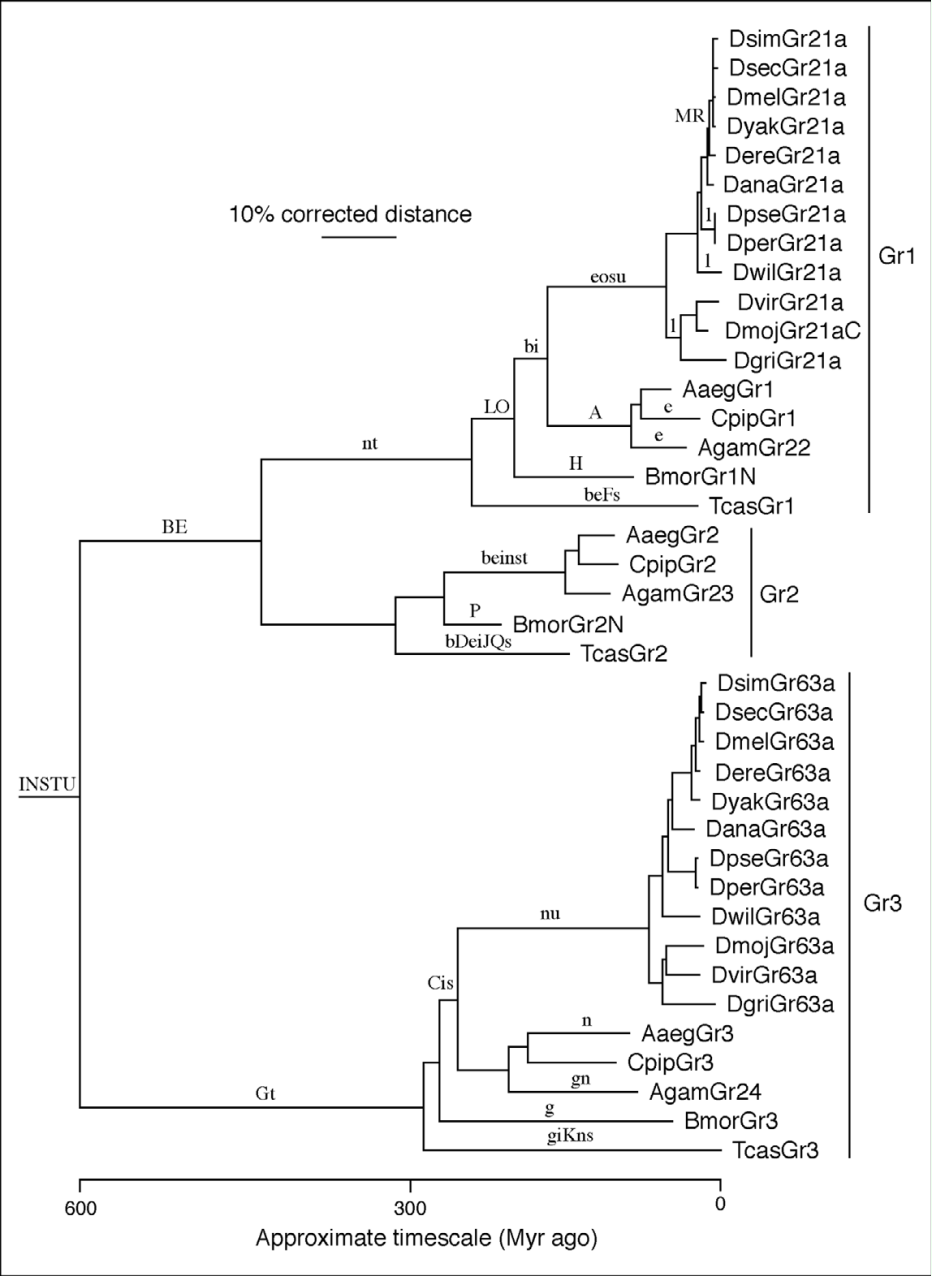
Phylogenetic relationships of the Gr 1-3 proteins in the twelve drosophilid flies, three mosquitoes, the silk moth, and the flour beetle. This is a corrected distance tree so cummulative distances along the longest branches total as much as 185%, while the actual distances are never larger than 78%. Species names are abbreviated to the first letter of the genus in capitals and the first three letters of the species name in lower case. The extreme N-termini of the BmorGr1 and BmorGr2 proteins could not be identified in the genome sequence, indicated by the suffix N after the protein name, but this does not affect the alignment used here. Similarly, a few amino acids are missing from the DmojGr21a C-terminus. Bootstrap support from 1000 replications of full heuristic uncorrected distance analysis was over 75% for all nodes except within the drosophilid flies. Inferred intron gains and losses are indicated by upper and lower case letters, respectively, on the relevant branches. Note that intron t, although only present in the Gr2 lineage, is inferred to be a much older intron because it is shared by other Grs (see Figure 2 legend).

In addition to the conserved orthologs of DmGr21a and DmGr63a in *An. gambiae* (AgGr22 and AgGr24), the *An. gambiae* genome contains a sister gene for DmGr21a/AgGr22, named AgGr23 (Hill et al. 2002). Like the DmGr21a/AgGr22 and DmGr63a/AgGr24 proteins, this third gene/protein lineage is also highly conserved in the yellow fever mosquito *Aedes aegypti* (Kent et al. 2008), the house mosquito *Culex pipiens* (HMR unpublished results), the silkmoth *Bombyx mori* (Wanner and Robertson 2008), and the red flour beetle *Tribolium castaneum* (Tribolium Genome Sequencing Consortium 2008) (Figure 1). Although Lu et al. (2007) include the Bombyx and Tribolium proteins in their supplementary material and name them BmGr22-24 and TcGr22-24, we feel this naming convention is awkward, and propose to name these genes Gr 1-3 in each of these species, in recognition of the fact that they are the only highly conserved and strictly orthologous lineages of Grs across these insects that are as old as 270 million years. We hope that this naming convention will be employed in future identifications of these three genes in non-drosophilid insects to minimize the nomenclatural confusion already engendered by their naming in *D. melanogaster* by cytological location (Clyne et al. 2000; Scott et al. 2001; Dunipace et al. 2001; Robertson et al. 2003) and in *An. gambiae* according to their order of discovery (Hill et al. 2002). Alignment of these three proteins from all these insects reveals that while their N- and C-terminal regions have different lengths and rather divergent sequences, the core transmembrane regions are well conserved and fully alignable, with at least 25% amino acid identity. The only minor length differences are in five of the intra- and extra-cellular loops between the trans-membrane domains (the last, apparently intra-cellular loop, between TM6 and TM7 does not vary in length). The first and last TM domains are the most highly conserved in amino acid sequence, as is true for the entire Gr family (Clyne et al. 2000). From the results of Lu et al. (2007) who expressed all three proteins in an “empty neuron” system in *D. melanogaster* antennae, it appears that all three contribute to the detection of CO_2_ in other insects.

**Figure 2.**
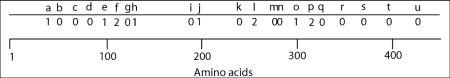
Schematic representation of intron locations in the Gr 1-3 genes. Introns were numbered from a-u and their positions are indicated relative to the ∼450 amino acid length of these proteins. This numbering is different from that in Robertson et al. (2003) — introns u, v, and c' therein for DmGr21a are introns l, m, and r here; introns f and m therein for DmGr63a are introns c and g here; and ancestral introns 1, 2, 3 therein are introns s, t, and u here. Intron phases relative to codons are shown below the intron names (0 is between codons; 1 is after the first base of a codon; 2 is after the second base of a codon).

### Intron evolution

As noted above, the intron content of the Gr21a gene lineage in the drosophilid flies is unusual. We attempted to place these introns in a broader context by considering the presence and absence of introns across all three gene lineages in all of these insects. Remarkably there are 21 different intron placements in the coding regions of this set of three genes (Figure 2; Appendix). It seems unlikely that the common ancestor of these three genes contained all 21 introns, indeed many are quite close to each other (e.g. introns m and n are one codon apart). Inference of intron losses and gains is clearly evident within many gene lineages, both when traced through species history (e.g. Roy and Gilbert 2005), and when traced in large paralogous gene families (e.g. Robertson 1998, 2000; Roy and Penny 2007). Indeed when considering the molecular evolution of the entire chemoreceptor superfamily as represented in *D. melanogaster*, Robertson et al. (2003) concluded that ∼57 introns gains had occurred in the superfamily and ∼48 intron losses were postulated to explain the current distribution of introns in the ∼120 genes.

Mapping of the intron locations on the phylogenetic tree of the genes in Figure 1 leads to the inference that the common ancestral gene lineage had at least five introns. This is two more than was inferred for this branch in Robertson et al. (2003), suggesting that the inference therein of just three ancestral introns for the entire superfamily was an underestimate. Indeed, given the 2-3-fold excess of intron losses over intron gains (see below), introns b, e, and g might also be ancestral to the gene lineage and subsequently lost in the Gr3 or Gr1/2 lineages, respectively. If b, e, and g are indeed older then the common ancestor gene would have had eight introns roughly equally spaced along its length. Thirteen intron gains (conservatively assuming that b, e, and g are older) are postulated to explain the current distribution of introns in these three genes, including most remarkably the two novel introns present in the Gr21a genes in the *melanogaster* subgroup species (introns m and r). One of these (m) is just one codon apart from intron n, and an alternative explanation might be that these are the same intron which has “slid” one codon in this highly conserved region of the gene/protein, however this model would require postulation of seven independent losses of the intron in the Gr21a lineage from beetles up to the *melanogaster* species grouping, which seems unlikely. Nevertheless, the complete absence of introns from this lineage in most of the drosophilids requires postulation of three independent losses of intron 1.

Recent intron gains are unusual in animal genomes (e.g. Roy and Gilbert 2005). This three-gene lineage appears to have acquired at least 13 introns in the past ∼600 Myr, for a rate of approximately 2.5 per billion years across all gene and species lineages (a total of roughly 5 billion years of evolution). Given an average coding length of 1200 bp, this is roughly 2 × 10^-12^ gains per possible insertion site per year, which is within the range of rates calculated by Roy and Gilbert (2005). The two most recent gains of introns m and r in the Gr21a gene at the base of the *melanogaster* species group are nevertheless at least 10, and as much as 20, Myr old (the time between the split of *D. ananassae* and the split of *D. yakuba/erecta*), and their short sequences of 53–57 bp are only weakly similar across these five species and bear no convincing similarity to other sequences in these genomes that might indicate their origin. Unfortunately the rapid rate of neutral evolution in these short-generation flies has obliterated any hint of the origins of these introns, and even global analyses of intron evolution in these fly genomes do not reveal additional intron gains whose origin is discernable (Coulombe-Huntington and Majewski 2007).

**Figure 3.**
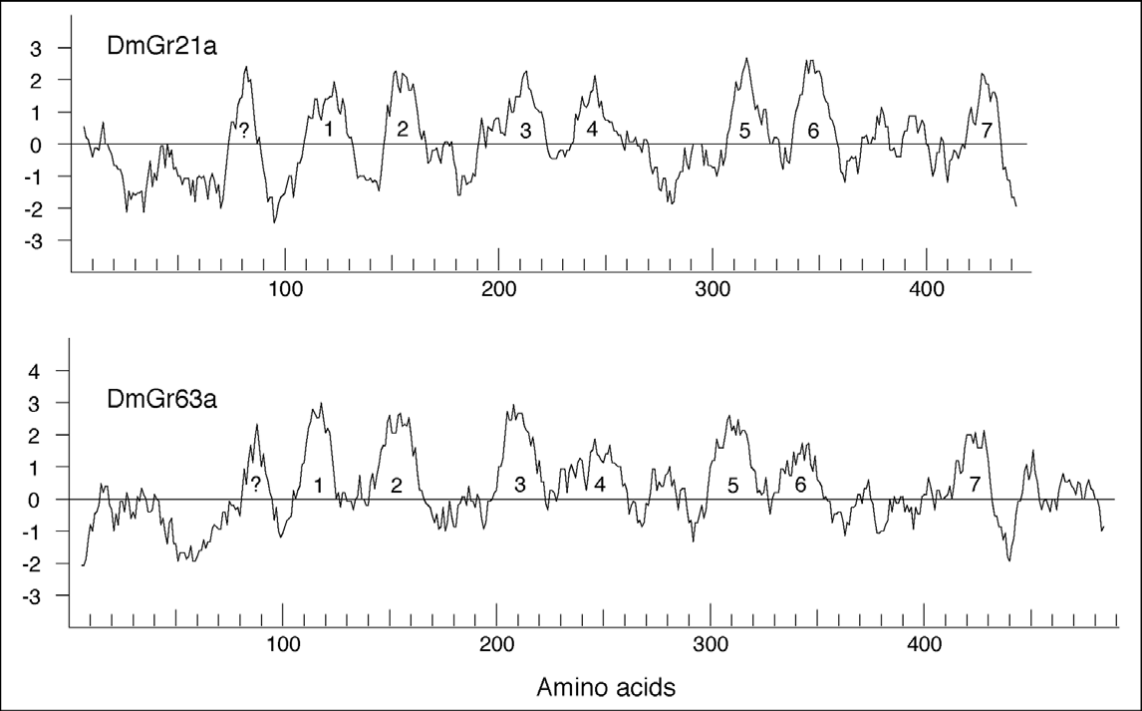
Kyte-Doolittle hydropathy plots for DmGr2la and DmGr63a. The seven transmembrane domains that align well throughout the Gr and Or families are numbered and the potential eighth TM domain at the N-terminus is indicated with a question mark.

At least thirty-nine intron losses are postulated on the tree, in keeping with many other estimates of considerably higher rates of intron loss than gain (e.g. Roy and Gilbert 2005; Roy and Penny 2007). The roughly equal rates of intron gain and loss estimated across the entire superfamily in *D. melanogaster* (Robertson et al. 2003) is likely to have resulted from underestimates of intron loss given the poor resolution of the superfamily tree and failure to recognize that several more introns might be ancestral to the superfamily. There appears to be a considerably higher rate of intron losses on the branches leading to the dipteran and beetle genes, with just one loss ascribed to a moth gene lineage. This bias towards intron loss in the dipteran and beetle genomes is consistent with their smaller genome size, and has been noted in genome-wide comparisons for the Diptera (e.g. Raible et al. 2005). As a result, no gene retains all five (or eight if b, e, and g are older) ancestral introns.

### Secondary structure

The insect chemoreceptor proteins had been considered to be members of a novel class of seven-transmembrane (7TM) G-protein-coupled receptors (GPCRs) (e.g. Hill et al. 2002; Benton et al. 2006), however their complete sequence divergence from all other known 7TM GPCR classes and their apparent functioning in extremely heterologous expression systems, e.g. frog oocytes (e.g. Wanner et al. 2007), has suggested that they might be a completely different class of membrane proteins. This possibility was supported by the finding of Benton et al. (2006) that the membrane topology of two odorant receptors is the reverse of that expected of a GPCR, with the N-terminus intracellular, a result confirmed for DmOr83b by Lundin et al. (2007). Wistrand et al. (2006) came to a similar conclusion, finding that the insect Ors and Grs do not have a membrane topology typical of all the other GPCR classes, indeed using the “positive-inside” rule of von Heijne (1989) they find that they likely have the opposite membrane polarity of the GPCRs. Benton et al. (2006) and Wistrand et al. (2006) did find that most *Drosophila* Ors have seven predicted TM domains, but some have eight. Kyte-Doolittle hydropathy plot analysis of many members of the Gr family, including DmGr21a and DmGr63a (Figure 3), reveals that they may have eight transmembrane domains, most of which are also recognized as potential TM domains by several TM prediction programs, including DAS-TMfilter (Cserzo et al. 2002) and PolyPhobius (Käll et al. 2005) with the first and last candidate TM domains being somewhat equivocal in the various Gr1-3 proteins. This ambiguity is particularly well displayed by the ConPredII server which uses the results of nine different prediction programs including the above two and predicts seven or eight TM domains for these proteins, with roughly half having the N-terminus inside the cell (Arai et al. 2004). If they indeed have eight TM domains, then the N-terminus could be internal yet the remaining membrane topology would be the same as GPCRs, however that would not fit with the findings of Wistrand et al. (2006) about the “positive-inside” rule. ConPredII calculates results for the “positive-inside” rule and for most of these Gr1-3 proteins strongly suggests the opposite topology to that of the GPCRs, in agreement with Wistrand et al. (2007). Resolution of this conundrum of the secondary structure and membrane topology of these insect chemoreceptors will require additional experimental study of both Ors and Grs.

**Figure 4.**
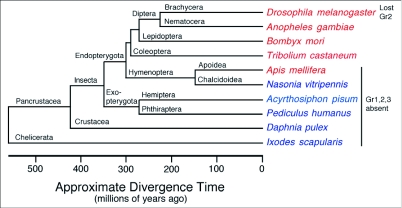
Phylogenetic relationships and approximate ages of the insects and related arthropods for which genome sequences are available. This figure is updated from the Honeybee Genome Sequencing Consortium (2006). Species in red are published and in blue have publicly available genome assemblies. Gene lineage absences are indicated on the right.

### Absence of this gene lineage from other available insect and arthropod genomes

This three-gene lineage is entirely absent from the genome of the honey bee, *Apis mellifera*, representing the more basal Hymenoptera (Robertson and Wanner 2006). It is possible that a draft genome sequence might be missing a few genes, perhaps because they reside in poorly cloned, sequenced, and assembled heterochromatic domains like the pericentromeric regions, however it is unlikely that all three of these genes would be absent from the draft genome assembly, which is of high quality (Honeybee Genome Sequencing Consortium 2006; Robertson et al. 2007). Furthermore, we searched all the raw traces from this genome using TBLASTN without finding a single read that encoded amino acid sequence with convincing matches to any of these three proteins. Honey bees have receptor neurons in their antennae that can detect carbon dioxide (Lacher 1964, cited in Winston 1991), and monitor its levels in their hives, responding to elevated levels by aerating the hive (e.g. Seeley, 1974).

Furthermore, examination of all other available basal insect and related arthropod genome sequences by TBLASTN searches of their publicly available draft assemblies, as well as all raw reads available in the Trace Archive at NCBI, reveals that this entire gene lineage is also missing from all of them (parasitoid wasp *Nasonia vitripennis*, pea aphid *Acyrthosiphon pisum*, body louse *Pediculus humanus*, waterflea *Daphnia pulex*, and blacklegged tick *Ixodes scapularis*) (Figure 4). The conservation of this protein lineage implies that it should be present in most of the above lineages, most of which are younger than 500 Myr (e.g. Glenner et al. 2006). Indeed, when an approximate timescale is plotted along the base of the tree in Figure 1, calibrated by the 250 Myr estimate for the split of the *Drosophila* flies and the mosquitoes within the Diptera, it appears that at least two, and perhaps all three, genes should be present in most or all of these arthropods. It seems unlikely that all three proteins independently became highly conserved only after the divergence of the Hymenoptera from the basal insect lineage. Instead this protein lineage likely exists in at least some basal insects and perhaps related arthropods, but was independently lost from each of the species currently targeted for genome sequencing.

There is little information on the ability of *Nasonia* wasps, *Acyrthosiphon* aphids, *Pediculus* lice, or *Daphnia* waterfleas, or related species, to detect carbon dioxide (but see Stross 1971). Indeed the biology of each of these arthropod groups suggests that they might no longer need to detect carbon dioxide and simply lost these receptors. However, like other ticks the blacklegged tick uses CO_2_ as a cue for the presence of vertebrate hosts (e.g. Holscher et al. 1980; Schulze et al. 1997; McMahon and Guerin 2002), so like honey bees they must use another method to detect carbon dioxide. This might involve other members of the chemoreceptor superfamily or it might involve a quite different mechanism like that reported in mammals (Hu et al. 2007) or in the gustatory system of *Drosophila* flies (Fischler et al. 2007). In either case it is remarkable that multiple methods of perceiving carbon dixoide appear to have evolved in arthropods.

## Appendix

Supplement. Amino acid sequences of the carbon dioxide receptor proteins.

>DmelGr21a MSFWAVSRGLTPPSKVVPMLNPNQRQFLEDEVRYREKLKLMARGDAMEEVYVRKQETVDDPLELDKHDSFYQTTKSLLVLFQ MGVMPIHRNPPEKNLPRTGYSWGSKQVMWAIFIYSCQTTIVVLVLRERVKKFVTSPDKRFDEAIYNVIFISLLFTNFLLPV ASWRHGPQVAIFKNMWTNYQYKFFKTTGSPIVFPNLYPLTWSLCVFSWLLSIAINLSQYFLQPDFRLWYTFAYYPIIAMLNC FCSLWYINCNAFGTASRALSDALQTTIRGEKPAQKLTEYRHLWVDLSHMMQQLGRAYSNMYGMYCLVIFFTTIIATYGSISE IIDHGATYKEVGLFVIVFYCMGLLYIICNEAHYASRKVGLDFQTKLLNINLTAVDAATQKEVEMLLVAINKNPPIMNLDGYA NINRELITTNISFMATYLVVLLQFKITEQRRIGQQQA

>DsimGr21a MSFWAVSRGLTPPSKVVPMLNPNQRQFLEDEVRYREKLKLMARGDAMEEVYVRKQETVDDPLELDKHDSFYQTTKSLLVLFQ IMGVMPIHRNPPEKNLPRTGYSWGSKQVMWAIFIYSCQTTIVVLVLRERVKKFVTTPDKRFDEAIYNVIFISLLFTNFLLPV ASWRHGPQVAIFKNMWTNYKYKFFKTTGSPIVFPNLYPLTWSLCVFSWLLSIAINLSQYFLQPDFRLWYTFAYYPIIAMLNC FCSLWYINCNAFGTASRALSDALQTTIRGEKPAQKLTEYRHLWVDLSHMMQQLGRAYSNMYGMYCLVIFFTTIIATYGSISE IIDHGATYKEVGLFVIVFYCMGLLYIICNEAHYASRKVGLDFQTKLLNINLTAVDAATQKEVEMLLVAINKNPPIMNLDGYA NINRELITTNISFMATYLVVLLQFKITEQRRIGQQQT

>DsecGr21a MSFWAVSRGLTPPSKVVPMLNPNQRQFLEDEVRYREKLKLMARGDAMEEVYVRKQETVDNPLELDKHDSFYQTTKSLLVLFQ IMGVMPIHRNPPEKNLPRTGYSWGSKQVMWAIFIYSCQTTIVVLVLRERVKKFVTSPDKRFDEAIYNVIFISLLFTNFLLPV ASWRHGPQVAIFKNMWTNYQYKFFKTTGSPIVFPNLYPLTWSLCVFSWLLSIAINLSQYFLQPDFRLWYTFAYYPIIAMLNC FCSLWYINCNAFGTASRALSDALQTTIRGEKPAQKLTEYRHLWVDLSHMMQQLGRAYSNMYGMYCLVIFFTTIIATYGSISE IIDHGATYKEVGLFVIVFYCMGLLYIICNEAHYASRKVGLDFQTKLLNINLTAVDAATQKEVEMLLVAINKNPPIMNLDGYA NINRELITTNISFMATYLVVLLQFKITEQRRIGQQQS

>DereGr21a MSFWAVSRGLTPPSKVVPMLNPNQRQFLEDEVRYREKLKLMARGDAMEEVYVRKPETVDDPLELDKHDSFYQTTKSLLVLFQ IMGVMPIHRNPPEKNLPRTGYSWGSKQVMWAVFIYSCQTTIVVLVLRERVKKFITSPDKRFDEAIYNVIFISLLFTNFLLPV ASWRHGHQVAIFKNMWTNYQYKFFKTTGSPIVFPNLYPLTWSLCVFSWLLSIAINLSQYFLQPDFRLWYTFAYYPIIAMLNC FCSLWYINCNAFGTASRALSDALQTTIRGEKPAQKLTEYRHLWVDLSHMMQQLGRAYSNMYGMYCLVIFFTTIIATYGSISE IIDHGATYKEVGLFVIVFYCMGLLYIICNEAHYASRKVGLDFQTKLLNINLTAVDSATQKEVEMLLVAINKNPPIMNLDGYA NINRELITTNISFMATYLVVLLQFKITEQRRVGQQQP

>DyakGr21a MSFWAVSRGLTPPSKVVPMLNPNQRQFLEDEVRYREKLKLMARGDAMEEVYVRKPETVDDPLELDKHDSFYQTTKSLLVLFQ IMGVMPIHRNPPEKNLPRTGYSWGSKQVMWAIFIYSCQTTIVVLVLRERVKKFVTSPDKRFDEAIYNVIFISLLFTNFLLPV ASWRHGPQVAIFKNMWTNYQYKFFKTTGSPIVFPNLYPLTWSLCVFSWLLSIAINLSQYFLQPDFRLWYTFAYYPIIAMLNC FCSLWYINCNAFGTASRALSDALQTTIRGEKPAQKLTEYRHLWVDLSHMMQQLGRAYSNMYGMYCLVIFFTTIIATYGSISE IIDHGATYKEVGLFVIVFYCMGLLYIICNEAHYASRKVGLDFQTKLLNINLTAVDSATQKEVEMLLVAINKNPPIMNLDGYA NINRELITTNISFMATYLVVLLQFKITEQRRIGQQQQ

>DanaGr21a MSFWAVSRGGTPPSKVVPMLNPNQRQFLEDEVRYREKLKLMARGDAMEEVYVRKQETVDDPLELDKHDSFYHTTKSLLVLFQ IMGVMPIHRNPPVRNLPRTGYSWGSKQVMWAIFIYSCQTTIVVLVLRERVKKFVTSPDKRFDEAIYNVIFISLLFTNFLLPV ASWRHGPQVAIFKNMWTNYQYKFFKTTGSPIVFPNLYPLTWSLCVFSWLLSIAINLSQYFLQPDFRLWYTFAYYPIIAMLNC FCSLWYINCNAFGTASRALSDALQMTIRGEKPAQKLTEYRHLWVDLSHMMQQLGRAYSNMYGMYCLVIFFTTIIATYGSISE IIDHGATYKEVGLFVIVFYCMGLLYIICNEAHYASRKVGLDFQTKLLNINLTAVDAATQKEVEMLLVAINKNPPIMNLDGYA NINRELITTNISFMATYLVVLLQFKITEQRRITQSQA

>DpseGr21a MSFWAVSRGLTPPSKVAPMLNPNQRQFLEDEMRYREKLKLVARGDAMDEVYVRKQETVDDPLELDRHDSFYQTTKSLLVLFQ IMGVMPIHRNPPVKNLPRTGYSWGSKQVMWAIFIYSCQTTVVVLVLRERVKKFVTSPDKRFDEAIYNVIFISLLFTNFLLPV ASWRHGPQVAIFKNMWTNYQYKFFKTTGSPIVFPNLYPLTWSLCIFSWVLSIAINLSQYFLQPDFRLWYTFAYYPIIAMLNC FCSLWYINCNAFGTASRALSDALQTTIRGEKPAQKLTEYRHLWVDLSHMMQQLGRAYSNMYGMYCLVIFFTTIIATYGSISE IMDHGATYKEVGLFVIVFYCMGLLYIICNEAHYASRKVGLDFQTKLLNINLTSVDAATQKEVEMLLVAINKNPPIMNLDGYA NINRELITTNISFMATYLVVLLQFKITEQRRTNSQAA

>DperGr21a MSFWAVSRGLTPPSKVAPMLNPNQRQFLEDEMRYREKLKLVARGDAMDEVYVRKQETVDDPLELDRHDSFYQTTKSLLVLFQ IMGVMPIHRNPPVKNLPRTGYSWGSKQVMWAIFIYSCQTTVVVLVLRERVKKFVTSPDKRFDEAIYNVIFISLLFTNFLLPV ASWRHGPQVAIFKNMWTNYQYKFFKTTGSPIVFPNLYPLTWSLCIFSWVLSIAINLSQYFLQPDFRLWYTFAYYPIIAMLNC FCSLWYINCNAFGTASRALSDALQTTIRGEKPAQKLTEYRHLWVDLSHMMQQLGRAYSNMYGMYCLVIFFTTIIATYGSISE IMDHGATYKEVGLFVIVFYCMGLLYIICNEAHYASRKVGLDFQTKLLNINLTSVDAATQKEVEMLLVAINKNPPIMNLDGYA NINRELITTNISFMATYLVVLLQFKITEQRRTNSQAA

>DwilGr21a MSFWAVSRGLSPPGATSKVAPMLNPNQRQFLEDEMRLREKMKLMARGDTNAMDDMYLRKPETVDDPLELDKHDSFYQTTKSL LVLFQIMGVMPIHRNPPVKNLPRTGYSWTSRQVMWAIFIYSCQTTIVVLVLRERVKKFVTSPDKRFDEAIYNVIFISLLFTN FLLPVASWRHGPQVAIFKNMWTNYQYKFFKTTGSPIVFPNLYPLTWALCVFSWLLSIGINLSQYFLQPDFRLWYTFAYYPII AMLNCFCSLWYINCNAFGTASHALSDALQATIRGEKPAQKLTEYRHLWVDLSHMMQQLGRAYSNMYGMYCLVIFFTTIIATY GSISEIIDHGATYKEVGLFVIVFYCMGLLYIICNEAHYASRKVGLDFQTKLLNINLTAVDAATQKEVEMLLVAINKNPPIMN LDGYANINRELITTNISFMATYLVVLLQFKITEQRRSQNTSTTIT

>DmojGr21aC MSFWAVSRGLTPQGKVAPMLNPNQRQFLEDELRYREKLKMLAGGNAIDDVYVRKPDTVDDPLELDKHDSFYKTTKSLLVLFQ IMGVMPIHRNPPVRNMPRTGYSWRSKQVMWAVFIYSCQTTIVVLVLRERVKKFITSPDKRFDEAIYNVIFISLLFTNFLLPV ASWRHGPQVAIFKNMWTNYQYKFFKTTGSPIVFPNLYPLTYALCVFSWLLSIAINLSQYFLQPDFRLWYTFAYYPIIAMLNC FCSLWYINCNAFGTASHALSDALQATIKGEKPAQKLTEYRHLWVDLSHMMQQLGRAYSNMYGMYCLVIFFTSIIATYGSISE IIDHGATYKEVGLFVIVFYCMCLLYIICNEAHYASHKVGMEFQTKLLNINLTAVDTATQKEVDMLLVAINKNPPIMNLDGYA NINRELITTNISFMATYLVVLLQFKITEQRR

>DvirGr21a MSFWAVSRGLTPPGKVAPMLNPNQRQFLEDELRYREKLKMLAGGTTIEDGYVRKPDTVDDPFELDKHDAFYRATKSLLVLFQ IMGVMPLIRNPPVKNMPRTGYTWTSKQAMWAMFIYAIQTTIVVLVLRERVKKFITSPDKRFDEAIYNVIFISLLFTNFLLPI ASWRHGPQVAIFKNMWTNYQYKFFKTTGSTIVFPNLYTVTYVLCTSSWLLSIAINLSQYFLQPDFSLWYTFAYYPIIAMLNC FCSLWYVNCNAFGTASRALSDALQATIRGDKPAQKLTEYRHLWVDLSHMMQQLGRAYSNMYGMYCLVIFFTSIIAAYGSISE IIDHGATYKEVGLFVIVFYCMCWLFIFCNEAHFASRKVGLDFQTKLLNINLTAVDTATQKEVDMLLVAISKNPPTMNLDGFA NINRELITTNISFMATYLVVLLQFKITEQRRSNIA

>DgriGr21a MSFWAVSRGQTPPGKVAPMLNPNQRQFLEDEIRYREKLKMQEGGMSNVYVRKLETVDDPDELDKHDSFYQTTKSLLVLFQIM GVMPIHRNPKDKNLPRTGYSWTSKQVMWAIFIYSCQTTIVVMVLRERVKKFITSPDKRFDEAIYNVIFISLLFTNFLLPVAS WRHGHQVAIFKNMWTNYQYKFFKTTGSPIVFPNLLPLTYALCVFSWLLSIAINLSQYFLQPDFRLWYTFAYYPIIAMLNCFC SLWYINCNAFGTASHALSDALQDTIKGDKPAQKLTEYRHLWVDLSHMMQQLGRAYSNMYGMYCLVIFFTSIIATYGSISEIL DHGATYKEVGLFVIVFYCMTLLYIICNEAHYASEKVGLEFQTKLLNINLTAVDTATQKEVDMLLVAINKNPPIMNLDGYANI NRELITTNISFMATYLVVLLQFKITEQRRANLNV

>AgamGr22 MIHTQMEDAQYEIRHQVLNPNQRQQLEDRRRIKEQLHQLEQDNESPTHMYRRKLKIASDVNLLDQHDSFYHTTKSLLVLFQI MGVMPIMRSPKGVDMPRTTFTWCSKAFLWAYFIYACETVIVLVVARERINKFISTSDKRFDEVIYNIIFMSIMVPHFLLPVA SWRNGSEVAKFKNMWTDFQYKYLIVTGKPIVFPKLYPITWTLCIVSWSLSLVIILSQYYLQPDFQFCHTFAYYHIIAMLNGF CSLWFVNCTAFGTASKAFAKELTDVLATERPAAKLTEYRHLWVDLSHMMQQLGKAYSNMYGIYCLVIFFTTIIATYGSLSEI IEHGATYKEVGLFVIVFYCMSLLFIICNEAHHASKRVGLNFQERLLNVNLTAVDKATQKEVEMFLVAIDKNPPTMNLDGYAN INRGLITSNISFMATYLVVLMQFKLTLLRQSAKNAFISALKANLSRIRSLDADKVNT

>AaegGr1 MIHSQMEDSQYQIRQQILNPNQRQQLEDNRRIKEQMQQLQRDDASPSHMYIRKLEFQADVNLLDKHDSFYHTTKSLLVLFQI MGVMPIVRSPKGVNMPRTTFTWFSKAFIWAYFIYACETVLVVLVAKERIKRFISTSDKRFDEVIYNIIFMSLLVPHFLLPVA SWRNGSEVAKFKNMWTDYQYKYLMVTGKPIVFPKLYPITWVLCVVSWAVSFVIIMSQYYLQPDFQLTHTFAYYHILAMLNGF CSLWFVNCTAFGTASKAFAQELSNILATEQPADKLTEYRHLWVDLSHMMQQLGKAYSNMYGIYCLVIFFTTIIATYGALSEI IEHGATYKEVGLFVIVFYCMGLLFIICNEAHHASRRVGLNFQERLLNVNLTAVDKATQKEVEMFLVAIDKNPPTMNLDGYAN INRGLITSNISFMATYLVVLMQFKLTLLRQSARKALIPALRANLTKLKEN

>CpipGr1 MIHSQMEDAGYQIRQQVLNPNQRQQLEDNRRIKEQMEQLQKENASPTRLYLRKMKVQADVNLLDHHDSFYHTTKSLLVLFQI MGVMPIVRSPPGVNMPRTTFNWGSRAFIWAYLIYAIETVYVVLVAKERINKFISNSDKRFDEVIYNVIFFSIMVPHFLLPTA SWRNGAEVAKFKNMWTDYQYKYLVVTGKPIVFPKLYPITWALCVVSWGVSFAVIMSQYYLQPDFQLWHTFAYYHIIAMLNGF CSLWFVNCTAFGEASKAFAAELSNIFATDRPADKLTEYRHLWVDLSHMMQQLGKAYSNMYGIYCLVIFFTTIIATYGALSEI IEHGATYKEVGLFVIVFYCMSLLFIICNEAHHASKRVGLNFQERLLNVNLTAVDKATQKEVEMFLVAIDKNPPTMNLDGYAN INRGLITSNVSFMATYLVVLMQFKLTLLRQSAKKALIASLTTNLTNIAAAKTNPQ

>BmorGr1N DIYGPEITDKDDGALLDKHDSFYLNTKSLLVLFQIMGVMPIMRVPKSAQTTRRTTYNWISKATLWAYLVWGLECIIVVKVGQ ERLANFQIGSNKRFDEVIYNIIFLSILIPHFLLPIASWRHGPQVAIFKNMWTHYQLKYLKITGKPIVFPNLYILTWGLCIFS WVLSFAVVLSQHYLQDDFELWHSFAYYHIIAMLDGFCSLWYINCNAFGTASRGLAINLHKALEAEHPALKLAQYRHLWVDLS HMMQQLGRAYSNMYGIYCMVIFFTTTISLYGALSEILEHGLSYKEMGLFVIVAYCMTLLFIICNEAYHASRKVGHEFQDRLL NVNLGAIDRSTQREVEMFLVAIAKNPPIMNLDGFTNINRELFTANISFMSTYLIVLMQFKLTLLRQGARKTVTAIVRAIFNT TITDNGAGGSDEDQE

>TcasGr1 MRNDHGSNTHLHPDDAIRRAKIVKVAASPTSANPDEEPDPELLDRYDNFYQTTKSLLVLFQIMGVMPIERSGKGRTTFRWLS STSIYAYFIFGAETIFVTMVFKERLYLILRPGKRFDEYIYGIIFLSILIPHFLLPVAAWTNGTEVAKFKNMWTRFQLKYYQV TGTPIIFHNLTLITYSLCVISWAVGIGIMLAQYYLQADMLLWHTFGYYHILAMLNCLCSLWFINCTAKGRVAVWMCNNLHKA LESRNPAKILGAYRDLWVDLSHMMQQLGKAYSGMYSMYCLLILLTTIVASYGSVTEIMDQGISFKEAGLFMIAFYCMTLLYI ICNEGHHATRKMGPEFRERLLNVNLSAVDQKTRQEVHMFLMAIEKNPPIMNLNGYANVNRKLISSTVTSIATYLVMLMQFRL TLMRNAQLAARRAIANVSVSSGNTTMS

>AgamGr23 MVIKESEFDDSLGYALLRRDMGTVWDTAKDERMVNGTMDPELIQRAKERAVRAQLNSADGDTCETHDQFYRDHKLLLVLFRG LAVMPITRSVPGRITFSWRSAASIYAFCFYLVSTVIVLVVGYERIKVFQTTTKFDEYIYGILFVIFLVPHFWIPFVGWGVAK QVAIYKTMWGAFQVRYYRVTGTSLQFPHLKLLIVFLSIGCLVCAIVFLLSLSFLLEGFALWHTSAYYHIITMLNMNSALWYI NSRGIRVASSSLSRCFRQDVAIECTAAMISRYRFLWLNLSELLQALGNAYARTYSTYCLFMFVNITVAIYGALSEIIDHGFG FSFKEIGLIVDTVYCSTLLFIFCDCSHNATLQVAQGVQDTLLSINLLKVDQPTQKEIDLFIQAIEMNPAIVSLKGYAEVNRE LLTSSIATIAIYLIVLLQFKLSLISQQIPVEIIENVKLLQKQ

>AaegGr2 MVIKDSEFEDSLNYALLRGDMGTTWDINKDERMMNGTLDPELIQRAKERAIRAQLNSADGDTCELHDQFYRDHKLLLVLFRA LAVMPILRSSPGRITFDWRSWASIYAYCFYVVSTVIVLIVGYERLKILQDTKKFDEYIYGVLFIIFLVPHFWIPFVGWGVAK HVAVYKTMWGAFQVRYYRVTGTNLQFPHLKILIVMFSIGCLVCAIVFLLSLSFLLEGFALWHTSAYYHIITMLNMNSALWYI NCRGIRVASSSLSDRFRKDVAIECTAAMISQYRFLWLNLSELLQALGNAYARTYSTYCLFMFANITIAIYGALSEVIDHGFG FSFKEIGLIVDTVYCSTLLFIFCDCSHNATLQVAQGVQDTLLGINLLKVDHPTQKEIDLFIQAIEMNPAIVSLKGYAEVNRE LLTASIATIAIYLVVLLQFKLSLISQQMPIELMEIKHSHKG

>CpipGr2 MVIKDSDFDESLNYALLRGDMGAIWDTTKDQRLMNGTMNPELIQRSKERAIRAQLNSADGDTAETHDQFYRDHKLLLVLFRA LAVMPILRSSPGRITFNWRSWASIYAYCFYFLSTIVVLVVGYERIKVLQETKKFDEYIYGVLFVIFLVPHFWIPFVGWGVAK HVAVYKTMWGAFQVRYYRVTGTNLQFPHLKVLIVIFSIGCLICAIVFLLSLSFLLEGFLLWHTTAYYHIITTLNMSSALWYI NCRGIRVASSSLSDRFRKDVAIECTAAIISQYRFLWLNLSEMLQALGNAYARTYSTYCLFMFVNITIAIYGALSEVIDHGFQ FSFKEIGLIVDTVYCSTLLFIFCDCSHNATLQVAQGVQDTLLSINLLKVDLPTQKEIDLFIQAIEMNPAIVSLKGYAEVNRE LLTSSIATIAIYLVVLLQFKLSLISQQMPVDLLENLQKAHAH

>BmorGr2N KEQEQRDLLSSQDGDTCEIHDQFYRDHKLLLVLFRALAVMPITRSRPGTITFSWKSTATIYAVCFYIAATAVVLIVGYERIQ ILQSIKRFDDYIYAILFIVFLVPHFWIPFVGWGVAHQVAIYKTNWGKFQVRYYRVTGENLKFPNLKTLIVIISVGCLLLAVC FLLSLCALLDGFLLKHTSAYYHIITMINMNCALWYINCKAIKIASQSLSECFQRDVDIECSAQLIARYRYLWLNLSELLQSL GNAYARTYSTYCLFMFANITIAVYGALSEIVDHGFGFTFKEVGLFVDAAYCSTLLFVFADCSHKSTLKVAAGVQDTLLSIDV LAVDRPTQKEIDHFIQAIEMNPAFVSLKGYAHVNRELLTSAISMITIYLIVLLQFKISLPKEPHGTGQ

>TcasGr2 MEISDLAQLYGNELHIKQISKWLRGSARAQEIQKRSELDSKDGHVIDEHDQFFRDHKLLLVLFRVLGVMPIQRGEIGRITFG WTSIPMLYAYVFYVVTTVLVVLVGYERFDILLNKSKKFDEYIYSIIFIIYLIPHFFIPFVGWGVAYEVCDYKNSWGGFQLHY YKITGKNLQFPLLSTLIIIISLGCLILAVVFLLTLSALLEGFTLYHTTAYLHIITMINMNCALWYINCRAVGNASTALAESF QNDVDRNCSAYIIAHYRVLWLSLSDLLQKMGNAYARTYSTYSLFMMANITVAVYGFTSEIVDHGIRFSFKEIGLLVDSTYCL FLLFVFCDCSHQASLNIARRVQVTLLQVNLSQVDPATRKEIDIFLVAIQMNPPKVSLKGYTVVNRELVTASVATIAIYLIVL LQFKISLLNMRG

>DmelGr63a MANYYRRKKGDAVFLNAKPLNSANAQAYLYGVRKYSIGLAERLDADYEAPPLDRKKSSDSTASNNPEFKPSVFYRNIDPINW FLRIIGVLPIVRHGPARAKFEMNSASFIYSVVFFVLLACYVGYVANNRIHIVRSLSGPFEEAVIAYLFLVNILPIMIIPILW YEARKIAKLFNDWDDFEVLYYQISGHSLPLKLRQKAVYIAIVLPILSVLSVVITHVTMSDLNINQVVPYCILDNLTAMLGAW WFLICEAMSITAHLLAERFQKALKHIGPAAMVADYRVLWLRLSKLTRDTGNALCYTFVFMSLYLFFIITLSIYGLMSQLSEG FGIKDIGLTITALWNIGLLFYICDEAHYASVNVRTNFQKKLLMVELNWMNSDAQTEINMFLRATEMNPSTINCGGFFDVNRT LFKGLLTTMVTYLVVLLQFQISIPTDKGDSEGANNITVVDFVMDSLDNDMSLMGASTLSTTTVGTTLPPPIMKLKGRKG

>DsimGr63a MANYYRRKKGDAVFLNAKPLNSANAQAYLYGVRKYSIGLAERLDADYEAPPLDRKKSSDSTASNNPEFTPSVFYRNIAPVNW FLRIIGVLPIVRRGPARAKFEMNSASFIYSVVFFVLLACYVGYVANNRIHIVRSLSGPFEEAVIAYLFLVNILPIMIIPILW YEARKIAKLFNDWDDFEVLYYQISGHSLPLKLRQKAVYIATVLPILSVLSVVITHITMSDLNINQVVPYCILDNLTAMLGAW WFLICEAMSITAHLLAERFQKALKHIGPAAMVADYRVLWLRLSKLTRDTGNALCYTFVFMSLYLFFIITLSIYGLMSQLSEG FGIKDIGLTITALWNIGLLFYICDEAHYASVNVRTNFQKKLLMVELNWMNSDAQTEINMFLRATEMNPSTINCGGFFDVNRT LFKGLLTTMVTYLVVLLQFQISIPTDKGDSEGANNITVVDFVMDSLDNDMSLMGASTPSTTTVGTTLPAPIMKQKGRKG

>DsecGr63a MANYYRRKKGDAVFLNAKPLNSANAQAYLYGVRKYSIGLAERLDADYEAPPLDRKKSSDSTASNNPEFTPSVFYRNIAPVNW FLRIIGVLPIVRRGPARAKFEMNSASFIYSVVFFVLLACYVGYVANNRIHIVRSLSGPFEEAVIAYLFLVNILPIMIIPILW YEARKIAKLFNDWDDFEVLYYQISGHSLPLKLRQKAVYIATVLPILSVLSVVITHITMSDLNINQVVPYCILDNLTAMLGAW WFIICEAMSITAHLLAERFQKALKHIGPAAMVADYRVLWLRLSKLTRDTGNALCYTFVFMSLYLFFIITLSIYGLMSQLSEG FGIKDIGLTITALWNIGLLFYICDEAHYASVNVRTNFQKKLLMVELNWMNSDAQTEINMFLRATEMNPSTINCGGFFDVNRT LFKGLLTTMVTYLVVLLQFQISIPTDKGDSEGANNITVVDFVMDSLDNDMSLMGASTPSTTTVGTTLPAPIMKQKGRRG

>DyakGr63a MANYYRRKKGDAVFLNAKPLNSANAQAYLYGVRKYSIGLAERLDADYEAPPLERKKSSESTASNNPEFTPSVFYRNIAPVNW FLRIIGVLPIVRNGPARARFEMNSASFIYSVVFFVLLACYVGYVANNRIHIVRSLSGPFEEAVIAYLFLVNILPIMIIPILW YEARKIAKLFNDWDDFEVLYYQISGHSLPLKLRQKAVYIATVLPILSVLSVVITHITMSDLNINQVVPYCILDNLTAMLGAW WFLICEAMSITAHLLAERFQKALKHIGPAAMVADYRVLWLRLSKLTRDTGNALCYTFVFMSLYLFFIITLSIYGLMSQLSEG FGIKDIGLTITALWNIGLLFYICDEAHYASVNVRTNFQKKLLMVELNWMNSDAQTEINMFLRATEMNPSTINCGGFFDVNRS LFKGLLTTMVTYLVVLLQFQISIPTDKGDSEGATNITVVDFVMDSLDNDMSLMGVSTSTPSTTTAGTTLPPPIMKQKGRKG

>DereGr63a MANYYRRKKGDAVFLNAKPLNSANAQAYLYGVRKYSIGLAERLDADYEAPPLDRKKSSESTASNNPEFTPSVFYRNIAPVNW FLRIIGVLPIVRHGPARAKFEMNSASFIYSVVFFVLLACYVGYVANNRIHIVRSLSGPFEEAVIAYLFLVNILPIMIIPILW YEARKIAKLFNDWDDFEVLYYQISGHSLPLKLRQKAVYIATVLPILSVLSVVITHITMSDLNINQVVPYCILDNLTAMLGAW WFLICEAMSITAHLLAERFQKALKHIGPAAMVADYRVLWLRLSKLTRDTGNALCYTFVFMSLYLFFIITLSIYGLMSQLSEG FGIKDIGLTITALWNIGLLFYICDEAHYASVNVRTNFQKKLLMVELNWMNSDAQTEINMFLRATEMNPSTINCGGFFDVNRT LFKGLLTTMVTYLVVLLQFQISIPTDKGDSEGATNITVVDFVMDSLDNDMSLMGASTPSTTTAGTTSPPPIMKQKGRKG

>DpseGr63a MANYYRRKKDAVFHNAKPINSGNAQAYLYGVRKYSIGLAERLDADYQPPPSDRKKSSDSTGSNNPEFTPSVFYRNIAPVNWF LRIIGVLPIVRRGPARAKFEMSSASFVYSVVFFMLLACYVGYVANNRIHIVRSLSGPFEEAVIAYLFLVNILPIMVIPILWW EARKIAKLFNDWDDFEVLYYQISGHSLPLRLRQKALYIAIVLPILSVLSVVITHITMSDLNINQVVPYCILDNLTAMLGAWW FLICEAMSTTAHLLAERFQKALKHIGPAAMVADYRVLWLRLSKLTRDTGNAMCYTFVFMSLYLFFIITLSIYGLMSQLSEGF GIKDIGLTITALWNIGLLFYICDEAHYASVNVRTNFQKKLLMVELNWMNSDAQTEINMFLRATEMNPSTINCGGFFDVNRSL FKGLLTTMVTYLVVLLQFQISIPTDKGDSDGGTNITVVDMLMDSLGNDMTILSASSSTTTHSTATSSTTPPPTSAKHGRGHR G

>DperGr63a MANYYRRKKDAVFHNAKPINSGNAQAYLYGVRKYSIGLAERLDADYQPPPSDRKKSSDSTGSNNPEFTPSVFYRNIAPVNWF LRIIGVLPIVRRGPARAKFEMSSASFVYSVVFFMLLACYVGYVANNRIHIVRSLSGPFEEAVIAYLFLVNILPIMVIPILWW EARKIAKLFNDWDDFEVLYYQISGHSLPLRLRQKALYIAIVLPILSVLSVVITHITMSDLNINQVVPYCILDNLTAMLGAWW FLICEAMSTTAHLLAERFQKALKHIGPAAMVADYRVLWLRLSKLTRDTGNAMCYTFVFMSLYLFFIITLSIYGLMSQLSEGF GIKDIGLTITALWNIGLLFYICDEAHYASVNVRTNFQKKLLMVELNWMNSDAQTEINMFLRATEMNPSTINCGGFFDVNRSL FKGLLTTMVTYLVVLLQFQISIPTDKGDSDGGTNITVVDMLMDSLGNDMTILSASSSTTTHSTATSSTTPPPASAKHGRGHR G

>DanaGr63a MASYYRRKKPDAVFLNAKPLNSANAQAYLYGVRKYSIGLAERLDADYEAPPVDRKKSSDSTASNNPEFTPSVFYRNIAPVNW FLRIIGVLPIVRRGPARAKFEMNSASFVYSVVFYILLSCYVSYVANNRIHVVRSLSGPFEEAVIAYLFLVNILPIMIIPILW SEARKIARLFNDWDDFEVLYYQISGHSLPLKLRQKAVYIAIVLPILSVLSVVITHITMSDLNINQVVPYCILDNLTAMLGAW WFLICEAMSTTAHLLAERFQKALKHIGPAAMVADYRVLWLRLSKLTRDTGNALCYTFVFMSLYLFFIITLSIYGLMSQLSEG FGIKDIGLTITALWNIGLLFYICDEAHYASVNVRTNFQKKLLMVELNWMNSDAQTEINMFLRATEMNPSTINCGGFFDVNRS LFKGLITTMVTYLVVLLQFQISIPTDKGDSEGSTNITVADLLMDSLDNDMTLMGSTATTASTTRVSTSLAPPTTKSTRGRKG

>DmojGr63a MASYYRRKKPDMVFLNAKPINSGNAQAYLHGVRKYSIGLAERLDSDYIPPPNDGKRSSVSTIASNNPDFTPSVFYRNIAPVN WFLRIIGVLPMVRRGPSRAKFALNSAAFIYSVVFFMLLAFYVGYVANKRIHAVRSLSGPFEEAVIAYLFLVNILPIIVIPIL WWEARKIARLFNDWDDFEVLYYQISGHSLPLNLRQKAVYIAIVLPILSILSVVITHITMSDLNLNQVVPYCILDNLTAMLGA WWFLICEAISTTAYLLAERFQKALKHIGPAAMVADYRVLWLRLSKLTRDTGNALCYTFVFMSLYLFFIITLSIYGLMSQLSE GFGIKDIGLTITALWNIGLLFYICDEAHYASVNVRTNFQKKLLMVELNWMNSDAQTEINMFLRATEMNPSNINCGGFFDVNR TLFKGLLTTMVTYLVVLLQFQISIPTDKGDGDGNSNMTVVDLLMDSLSNDMTLLGAPSTVATPSTTTTAVPPTVNRSGRGRK G

>DvirGr63a MASYYRRKKADTVFLNAKPINSGNAQAYLQGVRKYSIGLAERLDNDYIPPANDKKRGSISTVGSNNPDFTPSVFYRSIAPVN WFLRIIGVLPIVRRGPSRAKFALNSAPFVYSVVFFVFLACYVGYVANNRIHIVRSLSGPFEEAVIAYLFLVNILPIIIIPIL WLEAKKIALLFNDWDDFEVLYYQISGHSLPLNLRQKAIYIAILLPILSVLSVVIIHITMSDFNLNQVVPYCILDNLTAMLGG WWFLICEAISTTAYLLAERFQKALKHIGPAAMVADYRVLWLRLSKLTRDMGNALCYTFVFMSLYLFFIITLSIYGLMSQLSE GFGIKDIGLTITALWNIGLLFYICDEAHYASVNVRTNFQKKLLMVELNWMNSDAQTEINMFLRATEMNPSNINCGGFFDVNR TLFKGLITTMVTYLVVLLQFQISIPTDKGDGAGNSNVTVADMLMDSLGNDMTLLGTPSSTLAPTPTTTPVGRSGRGRKG

>DwilGr63a MANYYRRKKPDAVFLNAKPINSANAQAYLYGVRKYSIGLAERLDSDYQPPPIERKKSTASTGSNNPEFTPSVFYRNIAPVNW FLRIIGVLPIVRRGPARAKFEMNSAAFFYSVVFFMLLACYVGYVANNRIHVVRSLSGPFEEAVIAYLFLVNILPIMIIPILW WEAKKIARLFNDWDDFEVLYYQISGHSLPLHLRQKALYIAIILPILSVLSVVITHITMSDLNINQVVPYCILDNLTAMLGAW WFLICEAMSNTAHLLAERFQKALKHVGPAAMVADYRVLWLRLSKLTRDTGNAMCYTFVFMSLYLFFIITLSIYGLMSQLSEG FGIKDIGLTITALWNIGLLFYICDEAHYASVNVRTNFQKKLLMVELNWMNSDAQTEINMFLRATEMNPSNINCGGFFDVNRS LFKGLLTTMVTYLVVLLQFQISIPTDKGDGEASTNVTVVDMLMDSLDNDMTLLGPTSTAGTTATMRAAATTTTMATPTVKQG RAGRRG

>DgriGr63a MASYYRRKKPDTVFLNANPINSSNAQAYLQGVRKYSIGLAERLDSGYQKPSNDRKRSSVTTVDSQSLGTFTPSVFYRNIAPV NWFLRIIGVLPIVRSGPSRAKFALNTAPFLYSVIFFTLLACYVGYVAKNRIHIVRSLSGPFEEAVIAYLFLVNILPVMVIPI LWWEARKIARLFNDWDDFEVLYYQISGHSLPLNLRQKAIYIAIGLPIISVLSVVIIHMTMSDLNLNQVVPYCILDNLTAMLG AWWFIICEAISTTAYLLAERFQKALKHIGPAAMVADYRVLWLRLSKLTRDTGNALCYTFVFMSLYLFFIITLSIYGLMSQLS EGFGIKDIGLTITALWNIGLLFYICDQAHYASVNVRTNFQKKLLMVELNWMNMDAQTEINMFLRATEMNPSNINCGGFFDVD RSLFKGLLTTMVTYLVVLLQFQISIPTDKGDGDGDGNANMTVIDLLMDSMNNDMTVVGHSSTTPGTTAAPTTTTNTTTTPVN RSGGRGRKG

>AgamGr24 MRIERSSVHEPKRNRNVFLDVKPIADDANVNVPPRQAARRNATVFNNRVGFPPLTPKEAFVDAVPADQTCMVFESSKPIYLV LRAIGVLPYTRLPSGGTAFVLASPSMTYCVLFFLLLTVYIAFILLNRIEIVRTLEGRFEESVIAYLFIVNILPILIIPLMWY ESRKVVSVVNGWVDFETVYRETTGRALELRLRTKAQVIAILLPILCSLSVAITHVTMVDFKLLQVIPYCVLDTITYMMGGYW YMACETLSITAKILAEDFQRALRHVGPAAKVSEYRSLWLRLSKLARDTGFSTCYTFTFICLYLFFIITLSIYGLMSQISDGF GVKDIGLAVTAFCSVGLLFYICDEAHYASFNVRTNFQKKLLMVELSWMNTDAQTEINMFLRATEMNPSSINLGGFFDVNRTL FKSLLATMVTYLVVLLQFQISIPDEPSAMLMHSNSSHS

>AaegGr3 MNLNQDPIQYINLNNNARTVFLDVKPIYNEEKRKVSNGFNNRIGFPPISSRRVFGLESEFNTRSDIVYGTTKPIYNVLRMLG VFPFSRPSPGVTLFACASPAMAYCGVLFVTLMAYVIYITILRVHIVRTLEGRFEEAVIAYLFIVNILPVLIIPLMWYETRKV SSLLNQWVDFEAIYRKTAGRELELSFRTKALLIAILLPVLSCLAVIITHVTMVEFQLVQVIPYCILDTLTYMMGGYWYMTCE TLSITANILAEDFQRALRHVGPAAMVSEYRSLWLRLSKLARETGSSTCYTFTFLCLYLFFIITLSIYGLMSQISEGFGIKDI GLAVTAFCSVGLLFFICDEAHYASFNVRTKFQKKLLMAELSWMNSDAQTEINMFLRATEMNPSSINLGGFFDVNRTLFKSLL ATMVTYLVVLLQFQISIPDDSSMLVMHNMTGSYRE

>CpipGr3 MSIFPDTLRYIEVEPDPKTRAVFLDAKPAYLDHQQHQNRQTTNGFGNRVGFAEGPPREAFGDGGVVIKSDIIYDSSKPIYNV LRLLGVFPFMRPTAGMTMFACASPAMAYSVVFLVVLTIYVVFIMISRIDIVRTLEGRFEEAVIAYLFIVNILPLIIIPLMWY ETRKVCNLLNNWVDFEVLYQKTAGRELALNLKNKSLLIAVLLPVLSCASVIITHVTMVEFQLVQIVPYCILDTLTYMMGGYW YLACETLSTTANVLAEDFQMALRHVGPAAMVSEYRSLWLRLSKLARDTGFSTCYTFTFICLYLFFIITLSIYGLMSQISEGF GIKDIGLAVTAFCSIGLLFFICDEAHYASFNVRTNFQKKLLMVELTWMNSDAQTEINMFLRATEMNPSSINLGGFFDVNRTL FKSLLATMVTYLVVLLQFQISIPDDPTAMLKQNSTAAH

>BmorGr3 MSFEIKNNFFRTSVPIPNGFPVQTEAKSKNKPIFLDVSPAPTPKVNSPNAIIPMKNNLIDPFINKDIIYENIKPVFMVLRIM GVLPLTRTTSGVNEFHFISPAMVYSLTVFIILVSYISYLSLHKVQIVRNSEGKFEEAVIEYLFTVYLFPLTVVPILWYETRK IANVLNGWVQFEVTYKQLSNRILPVKLYKKSLLIAIIIPILSTTSVIVTHVTMVHFKTSQIIPYVFLEILTYMLGGYWYLLC EILSLCANVLADDFQQALRHVGPAGKVAKYRALWLRLSKLARNTGVANCYTFTFVNLYLFLIITLSIYGLLSKISEGFGTKD IGLALTALCSVFLLFFICDEAHYASHNVRTNFQKKLLMVELSWMNTDAQTEVNMFLRATEMNPSQISLGGFFDVNRTLFKSL LATMVTYLVVLLQFQISIPDATQPEIPTNIDDHVQNITDTTTEASSPISTLMSAFAKRKND

>TcasGr3 MYHQDQAVSILGEAIPKRRSVFLESGVNSADSFKASKVGPAPPIKFINKSSTDKFGNGAIYEVLKPIYALMRIVGIFPIKNT EPGMFRVAPELLGYSVVVFVVVMGYIGFIEWDKVEIVRSQEGRFEEAVIDYLFTVYLLPIIINPLVLYEARKLANVVTDWVN FERIYYKLTKKKLSVFFGNKPVILTVVLPLLACGVMVVTHITMAHFKIIQVVPYCYINCLIYLIGGFWFMQCDVVGKVASQL AEDFQMALKHVGPSSQVADYRSLWMLLSKLIRDVGNASGYTVTFLCLYLFLIITLTIYGLLSQLQAGFSTKDIGLTINAGLA IFILYFICDEAHYASNCLRVQFQKKLLLVELSWMNDEAQQEINMFLKATEMSPTDISLVGFFDVNRNLFKSLLATMVTYLVV LLQFQISIPEEASPTNSTTITTQTPN
